# The role of active inference in conscious awareness

**DOI:** 10.1371/journal.pone.0328836

**Published:** 2025-12-04

**Authors:** Jonathan Edward Robinson, Andrew W. Corcoran, Christopher J. Whyte, András Sárközy, Anil K. Seth, Gyula Kovács, Karl J. Friston, Cyriel M. A. Pennartz, Giulio Tononi, Jakob Hohwy, TWCF: INTREPID Consortium

**Affiliations:** 1 Monash Centre for Consciousness and Contemplative Studies, Monash University, Melbourne, Australia; 2 Brain and Mind Centre, University of Sydney, Sydney, Australia; 3 Centre for Complex Systems, University of Sydney, Sydney, Australia; 4 Institute of Psychology, Friedrich Schiller University Jena, Jena, Germany; 5 Sussex Centre for Consciousness Science and Department of Informatics, University of Sussex, Brighton, United Kingdom; 6 Program on Brain, Mind, and Consciousness, Canadian Institute for Advanced Research, Toronto, Canada; 7 Queen Square Institute of Neurology, University College London, London, United Kingdom; 8 Cognitive and Systems Neuroscience, University of Amsterdam, Amsterdam, Netherlands; 9 Wisconsin Institute for Sleep and Consciousness, University of Wisconsin–Madison, Madison, Wisconsin, United States of America; RIKEN CBS: RIKEN Noshinkei Kagaku Kenkyu Center, JAPAN

## Abstract

Active inference, a first-principles framework for modelling the behaviour of sentient agents, is beginning to be applied in consciousness research. One hypothesis arising from the framework is that active inference is necessary for changes in conscious content. As one component of an extensive adversarial collaboration among competing theories of consciousness, active inference will be contrasted with two other theories of consciousness, neither of which posit that active inference is necessary for consciousness. Here, we thus present a Study Protocol designed to test the active inference hypothesis using a carefully controlled adaptation of the motion-induced blindness paradigm, where an ‘active’ condition with richer active inference is contrasted with a ‘passive’ condition. In the active condition, participants direct their gaze towards a target stimulus following its disappearance from consciousness, and report on its subsequent reappearance. In the passive condition, participants maintain central fixation, while the stimulus array is moved across the visual field (in a replay of the active condition based on eye-tracking data acquired during active trials). In two experiments, we plan to investigate target reappearance across active and passive conditions to evaluate the contribution of active inference to conscious awareness. Results will eventually be considered in the context of all the experiments conducted as part of the overall adversarial collaboration.

## Introduction

Predictive processing theories of brain function and behaviour have accrued widespread interest in the cognitive sciences over the past two decades [1–5]. More recently, predictive processing has been advocated as a systematic foundation for the development of theories of consciousness [6–9]. In this Study Protocol, we describe two experiments designed to test a novel theory of consciousness, specifically a theory of changes in conscious content, derived from *active inference* (AI-C), a first-principles formulation of predictive processing. This theory will be contrasted with two other theories of consciousness, as part of an international adversarial collaboration led by the INTREPID consortium – one of five such projects funded under the Templeton World Charity Foundation’s ‘Accelerating Research on Consciousness’ initiative.

Active inference provides a normative framework for understanding how self-organising systems sample their world to resolve uncertainty and occupy their characteristic states [9–12]. Active inference (and the process theories that accompany it) conform to the free energy principle as an account of adaptive, self-organised behaviour [13,14]. In this perspective, autonomous agents (such as humans) act in ways that can be described as minimising a variational bound on free energy (surprise or self-information) over time, given a (generative) model of the probabilistic mapping between sensory outcomes and the latent states causing those outcomes. Such agents infer latent states and, crucially, infer policies for how to selectively sample sensory data that conform to their expectations of future sensory input, thereby accumulating evidence for their model and thus their own existence (cf. self-evidencing; [15]). The active sampling of sensory outcomes specified by an inferred policy forms a broad category of agency, which can occur in motor action, such as eye or head movement, or mental action, such as decisions to allocate spatial or feature attention.

In contrast to other theories of consciousness [16], such as theories in the predictive processing family, for example *neurorepresentationalism* (NREP) [17,18], and theories of different types, for example *integrated information theory* (IIT) [19,20], *global workspace* [21,22] and *higher order thought theory* (HOT) [23,24], active inference implies that active sampling of the sensorium, under an inferred policy, is aimed at reducing uncertainty about the causes of (actual or expected) sensations. For example, sampling in the visual domain through eye movements or other movement, or through mental active inference, namely selective attention allocation, would occur based on beliefs about the information gain enacting such policies would afford. Uncertainty reduction through active inference manifests as belief updating, which is hypothesised to underlie changes in conscious perception. There is reason to believe that active inference is implicated in conscious perception, since action imbues perception with a first-person perspective, and because active inference can help elucidate many findings in the science of consciousness, and help resolve various challenges in the field (for review, see [9]).

The active inference theory of conscious content (AI-C, hereafter) states that active inference is necessary for changes in conscious content; that is, if there is a change in conscious content, then there is active inference, and, equivalently, if there is no active inference, then there is no change in conscious content [8,9,25–32]. In a slogan, “to see is to look” – what is consciously ‘seen’ can only change when actively ‘looked at’ or ‘noticed’. Here, active seeing includes mental decision-making and planning such as selecting a policy for covertly attending to spatial location or object features (with concomitant disattention to other locations or features). Notice that this theory is minimal in the sense that it does not specify the sufficient conditions for the generation of conscious experience, leaving it open if other factors than active inference are needed. This theory contrasts to the other theories in this adversarial collaboration, NREP and IIT; these theories do not posit decision-making and planning in the motor or mental domains as a necessary condition on changes in conscious experience; that is, these competing theories allow changes in conscious content without the actions described formally in terms of active inference (though the theories naturally operate with different kinds of neural activity). The competing theories do recognize a role for action and attention in shaping conscious experience, such that what we look at or attend to help shape what we become conscious of, but they will appeal to theoretical constructs outside their core theoretical commitments in order to accommodate how attention and motor activity influence consciousness.

As described, AI-C says that active inference is a necessary condition for a change in conscious perception. On a strong reading, this theory would be challenged if a stimulus can become consciously perceived in the absence of active inference. This basic active inference hypothesis relates to conscious content and what mechanism underpins a change from seeing something to seeing something else (or from seeing nothing to seeing something). A corollary of this minimal active inference theory relates to the fact that abolishing all active inference for a participant is very difficult or may be impossible without rendering them wholly unconscious. This is because active inference includes a very broad category of activity, essentially any policy-guided gain control (or precision control) anywhere in a system such as the human brain (noting that this leaves room for many types of activity that are not formally speaking policy-guided, such as interpretation, imagery, and generation of top-down predictions). The corollary specifies what the theory implies for cases where active inference is diminished to some degree but not abolished, leading to the hypothesis that with less active inference or less confident (i.e., precise) active inference, change in conscious content will be hindered, slowed down or prevented. This consequence of the theory is motivated by the precision of the beliefs over policies, where policies with precise expected outcomes will garner more evidence for the beliefs in question. For example, if a policy of passively fixating—i.e., suspending active inference—is contrasted with a policy of actively foveating to a specific target, then AI-C predicts with strong confidence that conscious content should be facilitated in the latter case. It is this consequence of AI-C that will be tested in the experiments described in this study protocol. The competing theories, NREP and IIT, make predictions in the same direction or predictions of no change, and with less confidence, since they do not stipulate policy-guided action as necessary for a change in consciousness, but acknowledge that action may change conscious experience.

### Current study

Here, we describe a set of two experiments that form one of four Study Protocols (to be published separately), each of which is developed based on predictions of one of three major consciousness theories being tested in this adversarial collaboration (AI-C, IIT, and NREP).

As described above, the test of AI-C’s hypothesis proceeds by curtailing active inference in one, ‘passive’ condition, and contrasting that with an ‘active’ condition in which active inference leads to the selection of a policy of action with high expected information gain, with the prediction being that the active condition facilitates conscious awareness of the target. We will pursue this idea in a motion-induced blindness (MIB) paradigm, where a stationary peripheral target is rendered invisible due to the motion of a global pattern. We will contrast active sampling (via overt eye movement) with passive sampling (with eyes remaining at fixation) where, crucially, participants are exposed to identical visual input and retinal activation, and other task, attentional and expectation-related factors are kept constant. To assess the key prediction from AI-C that changes in conscious content will systematically occur earlier in the active condition, post hoc reports will assess whether the passively processed changes to sensory input are perceived later than the actively sampled changes.

In this study, we employ two experiments designed to assess these MIB active-passive differences (For more specific operationalised predictions relevant to the three theories considered in the adversarial collaboration (IIT, AI-C, NREP), see the section Operational Hypotheses below):

*Experiment A: Do changes in conscious experience occur earlier in the active than the passive condition?* This experiment will test whether the duration of MIB differs across the active and passive conditions. If there is no difference between conditions, or if MIB persists longer in the active condition, then that is strong evidence against AI-C. If MIB persists for longer in the passive condition, this would predominantly support AI-C.

*Experiment B: Does action improve sensitivity to backwards-masked stimuli?* This experiment combines MIB with a type of backward masking of the target stimulus. If the target is backwards-masked to preclude perception in the passive condition—by removing the target as it approaches the foveal region of the visual field—AI-C predicts that the target will still be perceived when the same masking setting is applied in the active condition. If there is no difference between conditions, or if the passive condition results in improved perceptual recognition, this would be evidence against AI-C; otherwise, this will provide evidence that action facilitates conscious awareness, and in that sense will predominantly support the active inference prediction.

Both experiments will also be equipped with measures of autonomic and neuromodulatory (i.e., cardiac and pupil) activity to adjust for any differences in attentional set (e.g., arousal) that could be considered to confound condition-specific differences. Further, in primates, attentional selective sampling under normal conditions is typically associated with motor activity (e.g., ballistic saccades or microsaccades, as well as eye blinks and pupil dilation and constriction), and therefore this study will record not only the saccades in the active condition (for play-back in the passive condition) but also microsaccades, pupil diameter and blinks throughout, in order to quantify differences between two conditions (such as microsaccades when fixating on the relevant location in both the passive and action conditions).

In the context of this Study Protocol, the conception of attention under active inference plays an important role in the overall dialectic. In the literature, several studies have investigated whether attention, as traditionally conceived, might dissociate from consciousness (for a review, see [33]). This research might contribute to the interpretation of findings from the current study, since if there are indeed changes in conscious perception without the attentional, mental action as conceived by active inference, then active inference would not be necessary for consciousness. For active inference however, attention (understood technically in terms of policy-guided precision control) forms a much broader category than in the standard literature, such that any kind of precision control, including motor active inference and any kind of mental active inference, technically counts as attentional processing. Therefore, for AI-C, findings to date suggestive of a dissociation would need to be discussed in the light of this broader conception of attention. Here it is plausible that AI-C would indeed be consistent with these findings, since active inference may still be in play in consciousness even if vernacular conceptions of attention are not – though a full case to this effect would have to be made. In the context of the present adversarial collaboration, this dialectic would unfold in terms of critical comparative analyses of the active inference conception of attention and the favoured conceptions of attention generated from the theory leads of NREP and IIT.

### Current study in the context of adversarial collaboration

It is important to contextualise the significance of this test of AI-C in the broader setting of the INTREPID adversarial collaboration; this is because scientific expectations for adversarial collaborations can differ from more common scientific projects. An adversarial collaboration is designed to be able to distinguish among theories, rather than just test theories on their own; this implies that experiments are designed for which each theory has a prediction. Where theories are relatively diverse in their explanatory target and theoretical construction, as may happen in early (pre-paradigmatic) phases of scientific development, a quite diverse set of experiments may be designed, each of which test one theory more directly than the other theories. Thus one theory may make a clear and confident prediction about a given experiment, while other theories that are not directly tested may need to appeal to certain auxiliary hypotheses to generate a prediction. This can have the effect that predictions from some theories though directional are held with low confidence (due to the added complexity from adding auxiliary hypotheses). Furthermore, the effort to define tests for which all theories can have predictions can lead to compromises where the tests are not in fact strongly pertinent to the subject matter of the theories but instead relate to a more peripheral prediction (for example, it may be about a particular neuronal response rather than a finding in paradigmatic consciousness science).

The challenges associated with testing diverse theories can be mitigated by adopting a multi-experiment strategy whereby different experiments are more weighted towards key theoretical predictions of one theory than another. Taken together, these experiments will yield a balanced set of results pertaining to a range of predictions that are capable of arbitrating amongst the theories of interest. The overall outcome of the adversarial collaboration will then depend on discussion of these results in light of each theory lead’s preregistered predictions and their associated confidences (which serve to weight the relevance of a given experimental outcome for one’s theory), and the significance of any confounds and unforeseen elements. In this context, some empirical results may only lead to modest distinction among theories, while others may lead to more stark distinctions; however, the implications for each theory will not be clear until the findings of each experiment have been combined. It is thus important not to pass judgement on the outcome of an adversarial collaboration until all experiments have been concluded and their results fully integrated under a single, overarching analysis scheme. In addition to conceptual interpretations, a Bayesian adversarial collaboration analysis scheme can formally describe these scenarios, and provide numerical outcomes for each theory’s predictions (including the prediction confidences) across all experiments, and compare even disparate theories with each other (as long as each theory has some prediction about all experiments) (see [34], which also surveys the philosophy of science of adversarial collaborations).

Notice further that, in the logic of an adversarial collaboration, the aim is to distinguish among the involved theories. The benefit of this is that researchers in the field are then provided with an evidence-base for deciding which of those theories to believe the most, or to invest their own research time and resources in. A potential limitation is that the results may not necessarily be substantially informative for theories outside of the adversarial collaboration; for example, it could be that a further theory exists that would make overlapping predictions with one of the theories in the adversarial collaboration. Hence, from the perspective of such a further theory, the findings may not be of interest, even if they do strongly distinguish between the involved theories.

In the current adversarial collaboration (TWCF ARC INTREPID), three main experiments have been designed, one of which, described in this study protocol, is pertinent to AI-C. All three theories have predictions (with varying confidences) about each experiment, and the aspiration is that the outcome will distinguish one theory from the other two. The complete set of prediction tables have been made available in the preregistration [35], and we set out the details of the prediction tables for our experiment, and provide a context for these in a schematic for the entire set of predictions in the section Operational Hypotheses below. We refer the reader to the preregistration and the Study Protocols for details concerning the other experiments.

## Materials and methods

### Aim

**Fig 1:**
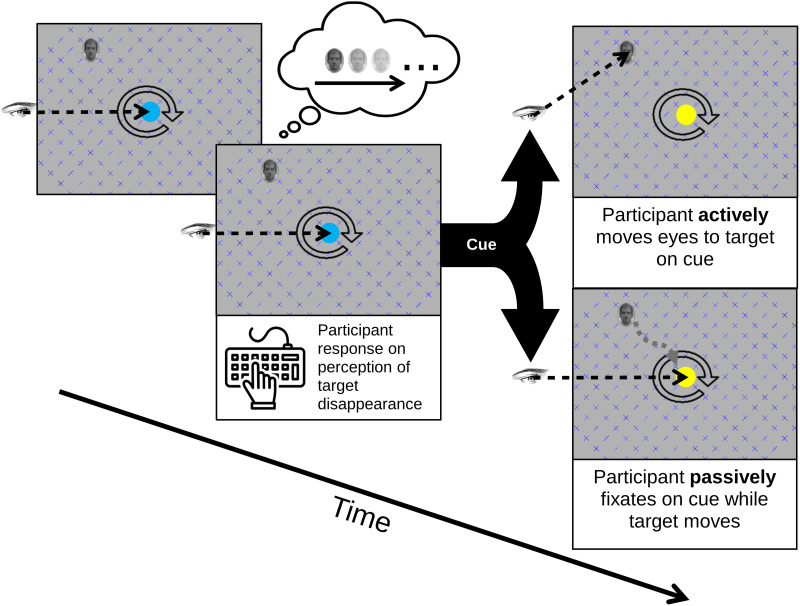
Schematic of trial timeline common to Experiment A and B. Thought bubble denotes perceptual (rather than physical) disappearance. The boxes after the forking cue arrows denote the distinction between the active (top) and passive (bottom) conditions. The circular arrow denotes motion of the gridded array (though its direction is randomly selected for each trial).

The aim of this study is to contrast passive and active conditions using peripheral targets in MIB. In the active condition—after the participant reports the initial disappearance (fading from awareness) of the stimulus—they will be cued to saccade to the previously observed location of the target. This saccade is considered as a manifestation of overt active inference. In the passive condition, following a reported disappearance, the sensory consequences of the saccade are ‘replayed’ by moving the stimulus array (which includes the grid), while central fixation is maintained. Passive replay will be based on eye movement trajectories recorded via eye tracking during the active condition, thereby ensuring that visual stimulation is identical across conditions. In each condition, participants will be instructed to indicate—via button press—when the target reappears in consciousness (see Fig 1).

### Status and timeline

In the latter part of 2020, two Templeton World Charity Foundation workshops were hosted online by Cyriel M. A. Pennartz, resulting in (1) the formation of the TWCF INTREPID consortium, (2) the selection and fleshing out of experimental paradigms capable of arbitrating amongst hypotheses derived from IIT, NREP, and AI-C. From this, the consortium initially commenced piloting, testing the viability and feasibility of the experiments in mediating between theories. In mid-2021 the experiment team at Monash University convened a meeting agreeing with Gyula Kovács from Friedrich Schiller University Jena to form the replication lab (impartial to the outcome) for the INTREPID consortium’s set of experiments testing the necessity of active inference for changes in conscious content. In late-2021 the Monash Lab ran piloting for proof-of-principle for the eye-tracking and behavioural components of the experiment. This design was then pre-registered on OSF (https://osf.io/35rhx) as part of the larger, multi-experiment structured adversarial collaboration (https://doi.org/10.54224/20646). Currently, piloting and paradigm development have been completed for both experiments and data collection is underway.

Based on this process, the experiments described herein form one of four protocols to be published, each describing experiments derived from predictions of the three theories being tested (AI-C, IIT, and NREP). Moving forward, consistent with the principles of a co-design, all amendments to the experiment protocol following this publication will be addressed by agreement between the teams at both Melbourne and Jena data collection sites, and the three INTREPID consortium theory leads (Karl J. Friston, Cyriel M. A. Pennartz, and Giulio Tononi). Each amendment will be documented in experimental reports identifying the specific change and rationale. A set of general analysis principles will be agreed upon prior to the commencement of the study; however, analysis pipelines will be developed and run separately in each lab to mitigate the potential for idiosyncratic analysis decisions to bias results. This process will be overseen by the Data Analysis and Replication Committee (DARC) of the INTREPID Consortium.

### Participants, sample size, exclusion/inclusion criteria, and stopping rule

Experiments A and B will be conducted in parallel at the Monash Centre for Consciousness and Contemplative Studies, Monash University (Melbourne, Australia) and the Institute of Psychology, Friedrich Schiller University (Jena, Germany). Identical stimulus materials and programme scripts will be used to run the experiments on identical setups and under very similar circumstances.

Participants will be students and staff recruited through each institution’s internal recruitment system. The general public may also be recruited through email approaches based on flyer responses. Participants in Melbourne will be reimbursed for their time either with course credit or AUD$20-50; participants in Jena will receive a similar rate in Euros or partial course credits. Both research sites have received ethical approval from their respective University Human Research Ethics Committees (Monash Human Research Ethics Committee – Ethics Number: 29238; The Ethics Committee of the Friedrich Schiller University – Ethics Number: FSV 22/093.

Recent MIB work deploying eye-tracking and pupillometry in the MIB paradigm [36] aimed for a target sample size of 55 participants to give a statistical power of at least 80% with an effect size of η2p = .13.; we will adopt this sample size as a stopping point for data collection. Participants must have normal (or corrected-to-normal) vision and audition, and will be aged between 18 and 45 years. Additionally, participants must demonstrate computer literacy and be able to maintain constant fixation for at least 5 seconds in the pre-test period and follow the fixation-based gaze strategy. Respondents will be excluded from participation if they (1) report having previously suffered a stroke or other neurological condition; (2) are currently taking psychoactive medications; or (3) are currently undergoing mental health treatment.

In Experiment A, participants will be excluded if they fail to report disappearances in more than 50% of trials or if they fail to generate a reappearance response in more than 25% of trials where they reported a disappearance. In Experiment B, participants will be excluded if they fail to reach the masking boundary at which there is stimulus reappearance <50% of the time, or in other words, if they fail to reach a threshold of stimulus awareness. Here ‘masking boundary’ describes a virtual circle, centred on the stimulus. If participants’ gaze enters this region, the stimulus will physically disappear. The same 50% rejection criteria will apply for reported disappearance and similarly participants who fail to complete > 25% of trials (including fixation, pre-cue period; post-cue period) for any reason will be excluded from data analysis. Furthermore, participants who do not meet the behavioural, eye tracking (e.g., microsaccade, eye-blink, and gaze location) exclusion criteria listed in the Data pre-processing section (below) will also be excluded from further analysis. Participant data collection will be considered complete when the total number of participants who surpassed these rejection criteria meets the stopping point defined above.

### Experiment A: Time to reappearance following motion-induced blindness

**Design, stimuli, and procedure.** This experiment deploys a within-subjects design to contrast differences in the time to reappearance of stimuli rendered subjectively invisible by MIB. The MIB task will be performed in conjunction with eye tracking in order to produce both active looking blocks and passive viewing blocks (where the active gaze is precisely replayed).

We will use the Racially Diverse affective expression (RADIATE) face stimulus set [37]. We use only neutral faces for our stimulus set, removing any stimuli with accuracy ratings for neutral emotional state below 80%. This leaves 108 stimulus images of distinct identities (and one used solely for instructional purposes). On each trial, the stimuli will be pseudo-randomly sampled with a constraint that no greater than two consecutive repetitions of a specific identity will be shown to participants. Each target stimulus will be converted to greyscale, scaled to 200x200 pixels, and luminance-matched using the SHINE toolbox [38]. All images will be enclosed in an elliptical mask centred on the face shape. In order to correct the luminance difference created by the mask the luminance of each stimulus image will be scaled to match an RGB value of 100 (+/- 0.1%); the background colour of the display will also be matched to this value.

Target stimuli will be presented at a randomly-selected polar angle (1–360 degrees) 9 degrees of visual angle from central fixation. In each trial, a blue central fixation dot will be present behind the stimulus and a cross pattern filling the entire screen will rotate at 2.7s/cycle, in line with previous work [39]. As the target in this design will either (in the passive case) remain stationary after being moved into central fixation or (in the active case) be foveated by the participant, it will ultimately fill the centre of the visual field in both conditions. This means that central attentional resources will eventually be allocated to it, and it will become visible even in the passive condition. This is consistent with active inference, since attention is a (covert) form of action. However, the thrust of this experiment lies in the role of overt action (eye movement), recognizing that covert attentional resources are also deployed in the passive condition at some point in the task.

Experiment A sessions will be separated into three sections:

*Training*. A crucial consideration—in both the analysis of eye tracking data and the use of eye tracking data to construct a retinal matched replay—is that epochs are not disrupted by additional saccadic eye-movements. Therefore in both experiments we will employ a training procedure similar to that reported in a study using fixation-event epoching by Kaunitz and colleagues [40]. Following standard calibration and validation procedures, participants will be taught to maintain their gaze on a central fixation dot, followed by a randomly positioned peripheral target (9 degrees visual angle). Initially, participants will only have to fixate on each target for 1 second, but after each trial the goal will be increased (+1 second) based on successful fixation, for a maximum of 5 seconds. As in previous work, this procedure should help participants to learn to pace their saccade and fixation rate so that they take time before changing their gaze. In this training phase participants will also be shown a warning if they fail to maintain their fixations for the target length. Participants will be given a maximum of 20 trials of 20 seconds (~6.6 minutes max total) to achieve this training standard. If the participant is unable to reach the 5 second maximum before the end of the training phase they will not progress to the next phase of the experiment. The last part of the training will use a two trial (20 seconds each; 40 seconds total) demonstration of the phenomena of perceptual disappearance using a physically removed stimulus, in order to teach the participant to differentiate between potential transient disappearances and full perceptual disappearance (occurring around 5–15 second from the start of exposure). Accounting for instruction time, this section takes a maximum of 10 minutes.

*Practice Experiment.* Following training, participants will be told how to complete active and passive trials before the main experiment begins. To ensure that participants fully understand the task, they will first perform a practice version of the experiment. This will be a short version of the main experiment that will allow participants to be walked through the task, under the supervision of the experimenter. The participant will complete six active and six passive trials, this practice will be repeated if the participant fails to successfully complete more than half the practice trials. If participants do not complete at least six trials (even including repeats) they will not progress to the next stage of the experiment. Participants will receive a warning during this phase on any trial where: 1) The minimum eye-gaze time for fixating in the centre of the screen was not met; 2) they look at the target when not instructed to.

*Main experiment*. Following the successful completion of training and practice trials, participants will be reminded (via diagram) how to complete active and passive trials before the main experiment begins. Participants will be informed that a cue in the active trial will allow them to move their eyes to look at the location where the target was previously seen; in contrast, in the passive trials the cue is an instruction to maintain fixation. During each experimental trial, the participant will be initially required to perform an uninterrupted fixation on a central dot for at least 1.5 seconds before responses can be given. If the participant is unable to maintain eye gaze on the target for more than 1.5 seconds, they will not be able to complete the trial. If the participant looks at the peripheral target stimulus during this time period, the trial will automatically end (and will not be counted as a valid trial). The participant will then be asked to press a button when the peripheral target stimulus entirely disappears; following this response, after a short delay (randomly jittered between 500ms-1000ms) a cue will be given to signal the next phase by flashing a different colour of the central fixation. In the active trial block, after the cue, the participant will be asked to look to the target location. In the passive block, participants will be told to maintain central fixation while the entire visual display (including the rotating grid) moves in relation to the fixation cross to eventually position, in an exact replay, the stimulus as it was in a randomly selected trial from the active block. Crucially, in this version of the experiment participants will be asked to respond as soon as they become aware of the target, thereby generating an estimate of the time to reappearance.

Each trial will last a minimum of 4 seconds (1.5 seconds for fixation; 500 ms pre-cue delay; 2 seconds post-cue) and a maximum of 20 seconds, with a randomly selected inter-trial-interval (ITI) ranging 1.5–2 seconds (during which only a central fixation dot will be displayed). If the maximum trial time is reached—without all elements of the trial being completed—this trial will be removed from further analysis. Each condition will comprise 72 trials with a break every 24 trials (a trial block). In each block, two catch trials will be included; these will appear pseudo-randomly (excepting the first and last 3 trials) in each block and will involve the physical disappearance of the stimulus. Note that these catch trials will end as soon as the participant reports awareness of this disappearance. Blocks of active and passive trials will be interleaved such that 24 active trials will be followed by 24 replay trials until 144 (+12 catch trials) trials of each type have been shown. Each image will be shown a maximum of four times in the active trials. Accepted active replays will be carried across passive blocks but the angle of their movement trajectory towards the centre of the screen will be randomised. This will ensure that stimuli can be replayed in the passive block without leading to systematic confounds relating to a specific movement trajectory.

### Experiment B: MIB target masking threshold for reappearance, content discrimination and confidence

**Fig 2:**
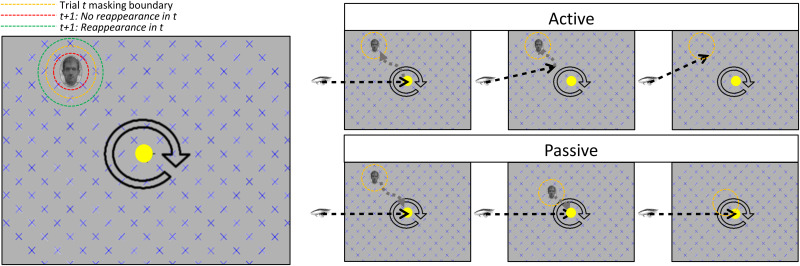
Diagram of the masking boundary in experiment B. All dashed lines here are invisible to the participant. In the left panel an orange dashed circle denotes the invisible boundary in a given trial (*t*). The red dashed circle denotes a decrease in the radius for trial *t + 1* as a consequence of a report of no reappearance in trial *t*. The green dashed circle denotes an increase in the radius for trial *t + 1* as a consequence of a report of a reappearance in trial *t*. In the right panel an orange dashed circle denotes the invisible boundary in a given trial (*t*). In active trials (top) the participant moves their eyes toward the stimulus and when their gaze hits the invisible boundary centred around the target, the stimulus disappears. In passive trials (bottom) the stimulus and the invisible boundary around it are moved towards the participant’s (centrally fixated) gaze. When the invisible boundary hits the participants’ gaze the target stimulus disappears. In the right panel the black dashed arrows denote the participants gaze and the grey dashed arrow denotes how the stimulus moves over the retina.

**Design, stimuli, and procedure.** Experiment B repeats the design of Experiment A with the (i) inclusion of a backwards masking procedure and (ii) the elicitation of stimulus discrimination and confidence reports. Stimulus masking will be initiated when the centre of gaze crosses an invisible circular boundary around the target stimulus. Whilst in the active condition the boundary will remain stationary around the target stimulus, in the passive condition the boundary will track the stimulus as it moves (via replay) towards the centre of the screen (see Fig 2 [right]). The radius of the circular boundary will be iteratively expanded or contracted using an adaptive staircase procedure designed to identify the threshold for conscious perception of the stimulus (see Fig 2 [left]). Additionally, in experiment B instead of immediately reporting reappearance, at the completion of the trial participants will be asked whether the stimulus reappeared.

Following each trial, if the participant reports a reappearance they will be required to discriminate the masked stimulus within a binary choice using either: the masked stimulus + a ‘no match’ option (where participants must identify that the stimulus matches the masked stimuli); or a foil drawn from the RADIATE stimulus set + a ‘no match’ option (where participants must identify that the foil does not match the masked stimulus). Half of all the choice arrays will contain the masked stimuli and half will not contain the masked stimuli. Each of the binary choices stimuli will be presented on randomly selected sides of the choice screen. Participants will make their choice by clicking on a slider using the mouse. Participants will be informed that where their cursor clicks from the centre of the screen (with the choices placed at the extremes of the slider) will be used to measure decision confidence, where clicks nearer to the ends of the slider indicate greater confidence. This choice screen is presented until the participant’s answer is confirmed with a button press.

*Training.* The training phase will be identical to that of Experiment A.

*Practice Experiment.* The practice phase will replicate that of Experiment A, with additional instruction about the stimulus discrimination task, the masking of the stimuli when making the saccades and the choice screen at the end of the trials. Participants will receive warnings if they incorrectly select foils during the stimulus discrimination task in order to encourage correct task engagement.

*Main experiment*. The main experiment follows the same general procedure outlined for Experiment A, with the additional inclusion of the backward masking, stimulus discrimination, and confidence report procedures. During active trials, participants will be cued to saccade to the target location after reporting its disappearance. When eye gaze crosses an invisible masking boundary, the stimulus will disappear. The precise radius of this boundary will be adjusted on a trial-by-trial basis (see Fig 2), such that the boundary will be expanded around the stimulus (i.e., masking is initiated earlier in the saccade) following trials in which the stimulus was reported to reappear, or the radius will be reduced around the stimulus (i.e., masking is initiated later in the saccade) following trials in which the stimulus was reported to have not reappeared. The number and length of trials will be identical with Experiment A.

### Data collection procedures

**Fig 3:**
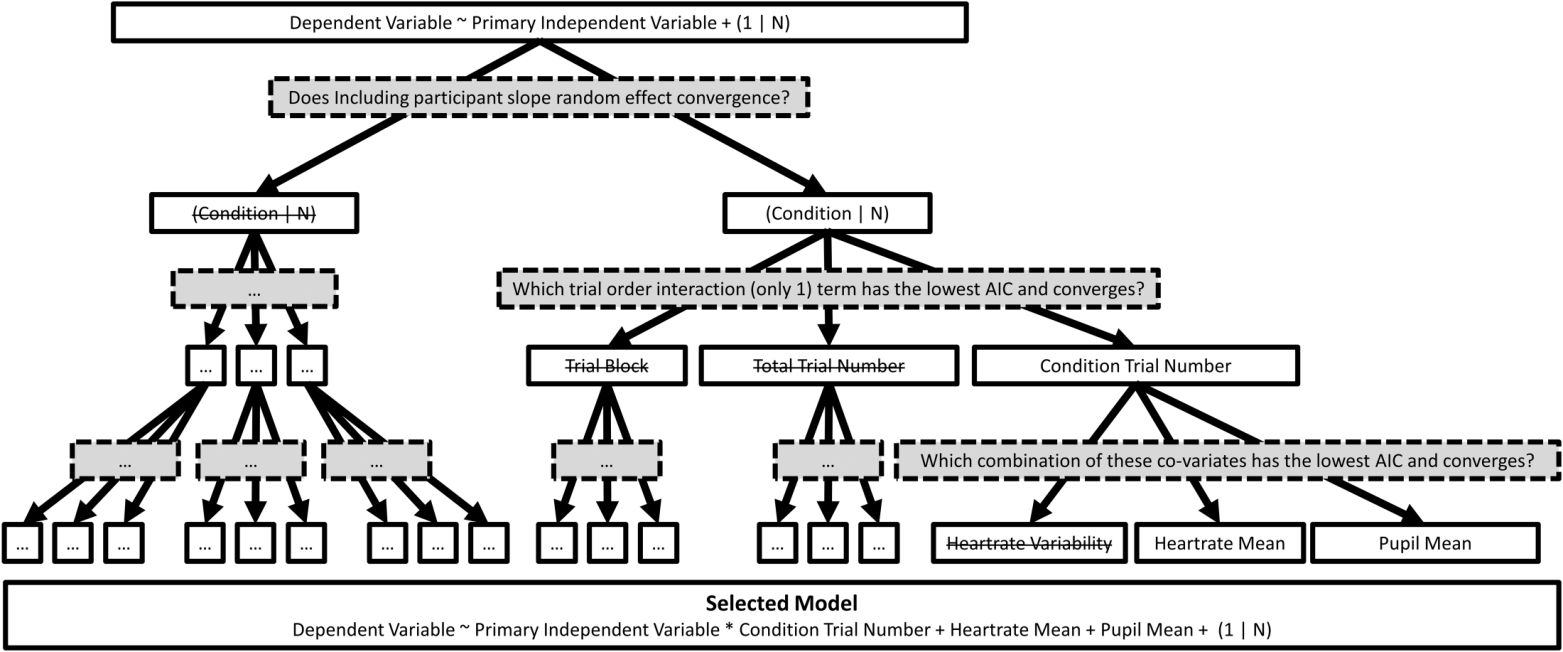
An example resolved LMM analysis decision tree for experiment A & B. Here solid lines denote variables to be considered and grey boxes (dotted lines) denote decision criteria. Note: 1) Crossed out options are for example only, all options are available in real analysis tree. 2) For display purposes only included variables are written in full but variable selection at each level includes the same forks. 3) The third level criteria uses ascending steps to decide model variable inclusion. 4) Top of the tree denotes the basic model.

**1. Behavioural Data.** Participants will use a key press to indicate the disappearance of the peripheral targets. A timestamp will be set when the disappearance response is given, when the cue is initiated, and when the stimuli are actively or passively foveated. This will allow precise extraction of replay gaze trajectories from the onset of fixation. In Experiment A, participants will be required to press the spacebar as soon as they become aware of the stimulus; in Experiment B, participants will be asked to give a forced binary choice after the trial to report whether stimulus reappeared. The invisible boundary that initiates the mask will be expanded if they report reappearance and contracted if they do not. If a reappearance is reported participants will be asked a subsequent question about the identity of the stimulus. The participant can choose a present identity or a ‘no match’ option. In 50% of the trials the identity shown in the trial will not match that presented in this post-trial question, allowing us to compute signal detection theoretic measures. Participant choice will also yield a confidence measure based on their click location (see Fig 3). To analyse the data we will fit linear mixed-effects models on the psychophysical awareness threshold. This will be quantified as the invisible boundary radius at which there is a 50% reappearance report rate under the model, with Active vs. Passive condition included as a predictor, potentially including and potential nuisance variables as covariates. Similar models will be fit to analyse signal detection theoretic measures of target discrimination and decision confidence.

Trials will be rejected if:

1) any button press response is made < 3 seconds following the start of the trial
2) time to reappearance is reported < 250 ms following the cue.
3) time to reappearance is reported > 1 second following the cue.

**2. Eye-tracking.** Binocular gaze and pupillary response data for the experiment will be sampled at a frequency of 1000 Hz using a desk mounted SR Research Eyelink 1000 system (35 mm lens). Testing will be conducted in a sound attenuated room and display luminance will be matched across recording sites (Melbourne and Jena) using luminance measures taken from the four screen quadrants, taking into account room lighting. Participants will be seated with their head stabilised on a chin rest mounted 105.3 cm from the stimulus presentation screen and ~55 cm from the eye-tracking camera to the eye. At the start of the experiment a nine-point calibration and nine-point validation will be conducted. An additional validation will be performed at the start of each block. If any inter-block validation is not considered ‘good’ (worst point error < 1.5°, average error < 1.0°), the initial calibration and block validation will be repeated.

***Passive condition, eye-movement replay.*** The replay data processing must be completed within the inter-block period and has therefore been kept relatively streamlined to prevent additional computation time:

1) Replay periods are epoched using a window extending from the initialization of the cue to the first fixation on the target stimulus.
2) Any values outside of the boundaries of the screen are removed, and eyeblink periods must be linearly interpolated between points in order to maintain a smooth replay. Any datapoint more than 1.5° of visual angle away from the linear path from fixation to target will not be considered in the replay. Any datapoint more than 1.5° away from the previous data point is considered an error and will not be considered in the replay.
3) Any trials containing poor signals (i.e., missing more than 20% of sample points in both eyes; [41]) will not be selected for replay. For replays that meet this standard all non-included samples will be linearly interpolated. To ensure equal numbers of active and passive trials, active trials may be used in replays additional times after running through all available replays. The replay path corresponding with the target stimuli will be used for replay if available, but the path trajectory will be transformed across randomly selected polar angles.

***Analysis.*** The following pre-processing steps describe the post-experiment analysis of eye tracking data for use in trial validation, eye-blink, microsaccade and pupillometry measures:

1) Trials in which participants fail to maintain 1.5 seconds of fixation within the allowable time period (20 seconds) are removed.
2) Any values outside the screen boundary are removed and eyeblink periods are linearly interpolated based on the surrounding data points. Eye blinks are defined by pupil size > 1.5 standard deviations below or 2 standard deviations above participants average pupil size.
3) Microsaccades will be quantified for each trial based on the microsaccade detection procedure defined by Engbert [42].
4) Trials are epoched using a window time-locked to the cue and first fixation on a target stimulus for both eye-gaze location and pupil size. The window therefore extends before these events (to encompass the fixation, cue, and subsequent movement) and after these events. In Experiment B, the window will be extracted time-locked to the cue and the onset of the mask.
5) Trials containing poor quality signals will be removed from subsequent analysis. Poor signal quality is defined as trials missing more than 30% of samples from both eyes [41]. Trials will also be rejected if the participant fails to fixate on a target stimulus or if the participant fails to maintain a central fixation in the passive condition during the period between the cue and the end of the movement sequence.

**3. Electrocardiogram.** To track physiological arousal during task performance, we will measure changes in heart period (i.e., inter-beat interval) derived from the electrocardiogram (ECG). We will acquire the ECG from a modified Einthoven lead-II configuration using either an ADInstruments PowerLab 26T device (MELBOURNE) or by a modified lead-I (clavicular) configuration using a BIOSEMI ActiveTwo EEG amplifier (JENA) and Biosemi flat active electrodes. Inter-beat intervals will be quantified as the duration between successive R-peaks (RR intervals). R-peaks will be identified using an automated QRS detection routine based on the Pan-Tompkins algorithm (e.g., [43]). Physiologically implausible inter-beat intervals (< 300 ms or > 2000 ms) will be rejected, as will any remaining intervals > 4 standard deviations from the participant’s mean. Missing intervals will be corrected via cubic-spline interpolation.

### Operational hypotheses

The hypotheses being tested by the experiments described in this Study Protocol are as follows:

Experiment A: changes in conscious experience manifest earlier in the active than the passive condition, as measured by behavioural responses;

Experiment B: backward masking can preclude changes in conscious experience in the passive condition but not (as much) in the active condition.

These hypotheses stem from the putative role of active inference to confirm the sensory input expected following action (as described in the Introduction). Active sampling should promote faster resolution of uncertainty, engendering earlier perceptual inference. These hypotheses can be expressed in terms of the following predictions about the patterns of data we expect to observe in each experiment:

1] *Subjective report of reappearance of peripheral stimuli will occur after a shorter delay following subjective disappearance in the active vs. the passive condition of Experiment A* (see Table 1).
2] *The psychophysical reappearance threshold for the radius of the masking boundary (once eye gaze is inside this invisible circular boundary the stimulus is masked) will be higher ; i.e., a larger radius in the active vs. the passive condition of Experiment B* (see Table 2).

**Table 1:** Predictions about the effect of active (vs. passive) sampling on target time-to-reappearance in Experiment A.

Theory	Decrease	No change	Increase	Confidence
IIT	X	X		Low
NREP	X	X		Medium
AI	X			High

**Table 2:** Predictions about the effect of active (vs. passive) sampling on mask onset boundary distance in Experiment B.

Theory	Decrease	No change	Increase	Confidence
IIT		X	X	Low
NREP		X	X	Medium
AI			X	High

Each of the other theories considered in the adversarial collaboration, IIT and NREP, offer different predictions to these, which also differ in their degree of confidence:

IIT is consistent with both manipulations (active or passive target sampling) quickly breaking suppression, by changing visual cortex state. Motor involvement (active) may be enough to tip the balance towards breaking suppression more easily, for a small difference in time to reappearance and/or masking boundary. Thus, for Experiment A (see Table 1), IIT predicts either decrease or no change, with low confidence; for Experiment B (see Table 2), IIT predicts either increase or no change, with low confidence.

NREP is compatible with motor movement conveying arousing/attentional effects (other than via active inference), which may facilitate early detection. For instance, motor activity can profoundly stimulate V1 firing activity, and motor-associated arousal may help to detect stimuli at greater distance from foveation point. Thus, for Experiment A (see Table 1), NREP predicts either decrease or no change, with medium confidence; for Experiment B (see Table 2), NREP predicts increase or no change, with medium confidence.

In addition to the two main hypotheses, we also predict the following, based on AI-C, which is not part of the adversarial collaboration and thus do not elicit predictions from the competing theories:

3) *In Experiment B target stimuli will be discriminated more accurately in active vs. passive trials given the same frequency of reappearance.*
4) *In Experiment B confidence in target discrimination will be higher in active vs. passive trials given the same frequency of reappearance.*

In the setting of the overall adversarial collaboration (TWCF ARC INTREPID), these sets of predictions will be considered in conjunction with predictions from all theories on three main experiments, in total. The aspiration is that this total set of findings from all experiments will strongly distinguish among these three theories (even if individual experiments are not able to strongly distinguish them). Table 3 illustrates some of the main predictions and confidences of each theory for selected, key parameters for each experiment, with Experiment 3 being described in this study protocol (for full details, please see the preregistration).

**Table 3:** Illustration of predictions and confidences for each theory about each consortium experiment (for selected, key parameters); for the full prediction tables and specification of the parameters, please consult the INTREPID preregistration.

Experiment	IIT	NREP	AI-C
1	Confirm prediction, high confidence	Predict no change, high confidence	Predict no change, medium confidence
2	Confirm prediction, medium confidence	Confirm prediction or predict no change, high confidence	Predict no change, medium confidence
3	Confirm prediction or predict no difference, low confidence	Confirm prediction or predict no difference, medium confidence	Confirm prediction, high confidence

### Planned analyses and expected outcomes

In order to capture the contribution of covariates within both experiments, 3A and 3B dependent variables will be analysed under a generalized linear modelling framework based on a pre-specified decision tree (see Fig 3). The choice of link-function will be informed by the distributional properties of the data and the evaluation of standard model diagnostics (e.g., Q-Q plot of residuals) and indices of marginal likelihood (e.g., AIC). Nested model comparison will be used to evaluate whether model fit is significantly improved by the inclusion of focal parameters. Random effects and additional covariates accounting for potential sources of noise (e.g., mean inter-beat interval, time-on-task) may be included if warranted by theoretical considerations and model comparisons (for a similar approach, see [44]). As part of this analysis we will quantify microsaccades frequency and latencies, as well as pupil area trajectory. These findings will be included in the results and, if we find that any of these data features, when considering the period immediately following the cue, differ significantly between conditions we will include these as covariates in the model.

### Data management and sharing plan

The management of intellectual property will align with the guidelines from the European Commission and the guidelines of both institutions involved in the project, as will our policy for sharing physiological, behavioural, and eye tracking data. Specifically, we will adhere to the European Commission’s open access and data management policy and the European Commission’s Grants Policy on sharing of unique research resources.

All investigators agree to willingly share data and materials associated with this project so as to expedite future discoveries in consciousness and maximise the study’s impact. Following the acquisition of data, all data and any subsequent analysis will be uploaded to a shared server available to all team members, as well as all members of the consortium and its associated committees. Over the life of the project analysis and findings based on these data will be shared through multiple media, such as (including but not limited to) media appearance, lab meetings, conference talks, and seminars (both at the host institutes and with the broader national and international scientific community).

In line with the principles of open science, de-identified data will be made available on an appropriate data repository. The specific items to be made publicly available as part of our resource sharing plan include:

1. All R/ Matlab/ Python code used for stimulus creation, manipulation, and experiment presentation, as well as all code used to pre-process or analyse the data.
2. All (de-identified and PHI removed) physiological, eye-tracking, and behavioural data adhering to FAIR data principles. Despite creating no neuroimaging data in this arm of the project, the larger TWCF project will be organised to align with the Brain Imaging Data Structure BIDS format (http://bids.neuroimaging.io). All metadata will be made available.
3. Any peer-reviewed publication (the TWCF ARC initiative mandates open access publications).

To allow the reuse of data—whilst also ensuring its integrity—downloading parties will be required to register before downloading any demographic, behavioural, eye tracking, or physiological data. A condition of the registration is that users agree: 1) not to distribute the data to third parties; 2) not to attempt to identify study participants; 3) to properly acknowledge the data resources. Data will be converted to shareable data formats (BIDS) and machine-readable annotations of the task and R/Matlab/Python scripts detailing the purpose of components will be included. There will be a GITHUB repository linked to the primary EBRAINS (or an equivalent alternative) repository that will be made available at the completion of the INTREPID project. We will review all data prior to upload to ensure they do not contain personal information or identifiable features. Data will be stripped of these details and will be General Data Protection Regulation (GDPR) compliant. We will share unprocessed (ASCII), minimally processed and final processed (CSV) data. All custom code and analysis pipelines will be shared. The experiment will be made available following publication via the OSF platform which links the user with all data being shared via EBRAINS (https://ebrains.eu/).

## Discussion

The study protocol addresses key behavioural commitments of a hypothesis about changes in conscious content derived from the active inference framework, contrasted in an adversarial collaboration with predictions derived from a predictive processing-based theory (NREP) and from Integrated Information Theory (IIT), neither of which posits action as a necessary condition on change in conscious experience. The Study Protocol therefore forms one of four Study Protocols (to be published separately) for this adversarial collaboration consortium (with two protocols for Experiment 2) as part of the INTREPID consortium project. Briefly, the prediction is that active inference—as enacted in overt eye-movement—will facilitate a change in conscious perception more than a passive condition, in a paradigm where a stimulus first made invisible through motion-induced blindness becomes consciously perceived. The contrasting, passive condition is where there is no overt action and where the change in conscious perception should be delayed or precluded.

An important factor, considered in the development of this study protocol, is the potential contribution of arousal in eliciting or promoting awareness, where arousal can take the shape of either phasic (stimulus-specific) or tonic (context-specific) arousal. In basic physiological terms, the role of arousal in psychophysical experiments such as those described here is well understood—specifically, that it enhances neural excitability and, depending on the level, may increase signal-to-noise ratios. Here, the locus coeruleus (LC) system mediates tonic and phasic arousal states affecting overall alertness, while phasic arousal helps fine-tune perception in response to specific stimuli. Studies using pupillometry have directly linked LC activity to changes in sensory processing, reinforcing the idea that arousal plays a major role in perception. All theories should thus expect these physiological processes to be in play, and understood as such the presence of arousal will not directly support one theory over the other. It will be a matter of further interpretation and modelling of the results to specify how arousal aligns with the core tenets of each theory. Here, it is of significance that, according to the active inference framework, arousal in this setting can be read in terms of attentional set and therefore reflects mental active inference, usually involving interoceptive inference [45–51]; this would make the arousal process an indispensable part of the active inference underpinning a change in consciousness. In contrast, for IIT and NREP, arousal would pertain to processes distinct from but interacting in important ways with the core processes posited by NREP and IIT as necessary for consciousness; this difference in outlook on arousal and attention partially explains the differences in the prediction table (shown above) for this experiment; that is, IIT and NREP’s predictions are not the opposite to those of AI-C, but partially overlap, and with weaker confidence. The interpretation of results, in particular differences of arousal between the two conditions, in the light of theoretical commitments will thus help situate the role of arousal and attentional set in diverse theories of consciousness. To help disambiguate the contributions of motor active inference (saccades) and mental active inference (i.e., attention and arousal) to perception, we opted to measure cardiac and pupil responses. Measuring inter-beat interval estimates allows us to adjust for context-specific arousal for a given trial. Pupillometric changes are considered integral to stimulus-bound aspects of active inference [51–53]. Cardiac and pupil data therefore allows us to quantify the contribution of (stimulus-specific and context-specific) attentional set and (saccadic) motor action to changes in perceptual content.

At a later stage of this overall project, the findings from this experiment will be considered in conjunction with further computational modelling and electrophysiological responses acquired with EEG or MEG (and further no-report iterations of these experiments). Computational modelling—under active inference—will elucidate the underlying computational mechanism giving rise to the hypothesised difference in behavioural responses and furthermore generate time resolved synthetic neuronal responses for comparison with empirical responses. EEG and MEG versions of the study will use frequency tagging techniques [54,55], which allows time-resolved tracking of stimulus-bound neural activity. Theoretically, such further grounding in computational modelling and neuronal data will address questions about the neurophysiological implementation of active inference in its own right (i.e., not in an adversarial setting) and, in particular, the coordination of motor action and mental action during active sensing. These studies will aim to distinguish top-down and bottom-up signals so as to characterise the time course of their propagation in the process of perceptual reappearance in motion induced blindness.

### The adversarial collaboration setting for this study

As mentioned in the Introduction, this study protocol forms part of a consortium project engaged in adversarial collaboration among theories of consciousness, in this particular case NREP and IIT, in addition to AI-C (https://doi.org/10.54224/20646). The results for the basic predictions described above, as well as the results from the other experiments in this adversarial collaboration, will be subject to discussion and comparative interpretation from the consortium, taking into account the overall set of predictions registered in protocols and at the OSF site. Furthermore, an integrative Bayesian analysis according to the scheme set out in [34] may be performed.

### Dissemination plans

The outcomes of this experiment, as with experiments from the two other theories (IIT and NREP) in the adversarial collaboration, will likely be published in one or more stand-alone articles in the first instance, or might be published in the form of a combined paper (i.e., individual experiments within each theory arm may or may not be published together within the same paper). The results of these experiments may be presented in posters and talks at both international and regional conferences.

Following the completion and publication of each set of experiments, additional papers aiming to integrate these findings and discuss their implications for each of the three theories tested in the adversarial collaboration may be published. For example, an integrative analysis along the lines of that proposed by Corcoran and colleagues [34] may be pursued.

Finally, the TWCF Consortium will deliver a public talk or symposium to present and discuss the outcomes of the entire adversarial collaboration.

### Dealing with amendments

In the present experiment and the two other protocols that form part of the INTREPID consortium, amendments will be considered and agreed upon between the teams at both Melbourne and Jena experiment sites, and the three INTREPID consortium theory leads (Karl J. Friston, Cyriel M. A. Pennartz, and Giulio Tononi). Upon the agreement of any reasonable amendments to the protocol, the amendment will be recorded and timestamped in the existing OSF preregistration documentation (https://osf.io/35rhx).
